# Extract from a Paris Letter, Dated November, 1866

**Published:** 1867-02

**Authors:** 


*Extract from a Paris letter, dated November, 1866*:—

"I have seen lately, through the kindness of M. Mathieu, the laryngeal speculum, invented by Dr. De Labordette of Lisieux.

"This instrument appears to be a happy modification of the ordinary laryngoscope which it simplifies greatly. It bears a resemblance to M. Cusco's vaginal speculum, and is composed of a lower and upper valve. This last plays upon the lower half, from which it can be removed to the distance deemed most convenient for exploration. When introduced into the throat, the superior valve becomes posterior. A peculiarity in its construction is its following a gradual curve, allowing this portion to be moulded upon the soft palate, and to descend into the pharynx. The anterior valve does not go beyond the root of the tongue, depresses this organ, and raises the epiglottis. The speculum, to be advantageously used, must be introduced as far as possible. When the instrument is in its place, the play of the valves, which is regulated by a handle held by the operator, lowers the tongue and maintains it in a stationary position. The posterior valve presents at its extremity a small, highly polished mirror, adapted to its inner surface. The upper orifice of the larynx is reflected upon this mirror, and the posterior portion of the epiglottis, the aryteno-epoglottidean folds, the laryngeal ventricle, as well as the inferior vocal chords, and the upper part of the trachea are distinctly visible. This exploration can be made in broad day-light, the only condition necessary being to seat the patient near an aperture. At night, an ordinary lamp or candle suffices. Before introducing the speculum, M. De Labordette recommends heating it over the flame of alcohol, or by immersion in warm water, as the chilly metallic surface produces a disagreeable sensation, and the breath tarnishes the mirror. The next step is to procure introduction very deeply. This part of the operation is easily effected without distress to the person examined. The instrument is closed when it enters the mouth. The valves are then made to separate, and the dilatation of the speculum is readily obtained. The laryngoscope is held with one hand, whilst the other applies caustics, etc., to those spots which appear diseased to the eye. In a descriptive pamphlet, Dr. De Labordette has published, he says that the play of the valves, in order to dilate the speculum, must only be attempted when the anterior segment has reached the base of the tongue, and that the patient must be told to breathe freely; otherwise, spasmodic contractions of the pharynx supervenes, and in consequence of this, exploration becomes very difficult, nay, impossible, owing to the great quantity of mucus secreted. From the rather bulky appearance of the apparatus, one would be inclined to think its introduction capable of occasioning distress or annoyance. This is not the case, however, and the instances in which a slight pain is noticed, are few in comparison with the number of times when the use of the speculum creates no unpleasant sensation. If it becomes necessary to inspect the larynx of a child, the instrument must immediately be made to enter to a considerable distance, and it should be kept in place, as the uncomfortable feeling soon yields before the reëstablishment of the respiratory functions. The absence of nausea is attributed to the size of the laryngoscope, which by uniform pressure over a large surface does not worry the palate. The tongue is completely mastered by the anterior valve, and its restlessness is kept in check. An advantage which M. De Labordette claims for his speculum, is that the buccal parietes are shielded from harm by the tubular construction of his instrument, this disposition permitting the use of medicinal agents with no more apprehension than the cauterization of the *os uteri* excites. Besides, it is possible to touch a very limited portion of the mucous membrane, avoiding thus easily the danger which was hitherto to be guarded against from action upon too extensive a surface. From this circumstance, as well as from the great facility for exploring the larynx, and even portions of the trachea, M. De Labordette was induced to try his invention in cases of laryngitis with production of false membranes. Five times, the introduction of the speculum allowed the application of caustics to be made with remarkable ease and accuracy, and the result was constantly favorable. It would be premature to pronounce upon the merits of this instrument from the small number of observations its inventor presents; still one cannot help being struck with the simplicity of M. De Labordette's speculum, which is so great that the process of introduction offers no difficulty, whilst permitting a very minute examination of the laryngeal parietes. This same laryngoscope has been employed lately to establish breathing in children who were born in a state of apparent suffocation."

---

SCABIES.—PARAFFINE.—Wash the parts well with a strong warm solution of common soda, and, after drying, apply paraffine very freely. A few repetitions will generally cure the case. —*Braithwait's Retrospect.*

